# Correlates of the incidence of disability and mortality among older adult Brazilians with and without diabetes mellitus and stroke

**DOI:** 10.1186/1471-2458-12-361

**Published:** 2012-05-17

**Authors:** Flávia Cristina Drumond Andrade, Pilar Egüez Guevara, Maria Lúcia Lebrão, Yeda Aparecida de Oliveira Duarte

**Affiliations:** 1 Department of Kinesiology and Community Health, University of Illinois at Urbana-Champaign, Champaign, USA; 2 Social and Cultural Anthropology, University of Illinois at Urbana-Champaign, Urbana, USA; 3 School of Public Health, Universidade de São Paulo, São Paulo, Brazil; 4 School of Nursing, Universidade de São Paulo, São Paulo, Brazil

**Keywords:** Diabetes, Stroke, Disability, Mortality, Brazil

## Abstract

**Background:**

The combined effect of diabetes and stroke on disability and mortality remains largely unexplored in Brazil and Latin America. Previous studies have been based primarily on data from developed countries. This study addresses the empirical gap by evaluating the combined impact of diabetes and stroke on disability and mortality in Brazil.

**Methods:**

The sample was drawn from two waves of the Survey on Health and Well-being of the Elderly, which followed 2,143 older adults in São Paulo, Brazil, from 2000 to 2006. Disability was assessed via measures of activities of daily living (ADL) limitations, severe ADL limitations, and receiving assistance to perform these activities. Logistic and multinomial regression models controlling for sociodemographic and health conditions were used to address the influence of diabetes and stroke on disability and mortality.

**Results:**

By itself, the presence of diabetes did not increase the risk of disability or the need for assistance; however, diabetes was related to increased risks when assessed in combination with stroke. After controlling for demographic, social and health conditions, individuals who had experienced stroke but not diabetes were 3.4 times more likely to have ADL limitations than those with neither condition (95% CI 2.26-5.04). This elevated risk more than doubled for those suffering from a combination of diabetes and stroke (OR 7.34, 95% CI 3.73-14.46). Similar effects from the combination of diabetes and stroke were observed for severe ADL limitations (OR 19.75, 95% CI 9.81- 39.76) and receiving ADL assistance (OR 16.57, 95% CI 8.39-32.73). Over time, older adults who had experienced a stroke were at higher risk of remaining disabled (RRR 4.28, 95% CI 1.53,11.95) and of mortality (RRR 3.42, 95% CI 1.65,7.09). However, risks were even higher for those who had experienced both diabetes and stroke. Diabetes was associated with higher mortality.

**Conclusions:**

Findings indicate that a combined history of stroke and diabetes has a great impact on disability prevalence and mortality among older adults in São Paulo, Brazil.

## Background

During the past decades Brazil has undergone demographic, epidemiological and nutritional transitions. One major effect of these transitions has been an increase in the prevalence of chronic non-communicable (CNC) diseases, particularly diabetes and stroke [1,2], and their subsequent impact on mortality and disability. Estimates for 2010 indicate that Brazil has the fifth largest population of individuals with diabetes in the world (7.6 million); this number is expected to rise to 12.7 million by 2030 [2]. A large share of diabetes cases (41%) is concentrated among Brazilians between the ages of 60 and 79 [2]. At the national level, calculations using data from the registry for diabetes and hypertension (SisHiperDia) indicated that 8.0% of registered diabetes cases reported having a previous stroke [1]. Even though this percentage is relatively small, the number of individuals with diabetes who have suffered a stroke is expected to increase in the coming decades, given the aging of the Brazilian population and the increasing prevalence of obesity and diabetes [1].

Currently, CNC diseases are responsible for 72% of deaths in Brazil [1]. Among these diseases, stroke and coronary heart disease are the main causes of death [3], and in 2002, diabetes was the fifth-leading cause of death in Brazil according to the World Health Organization. Mortality rates due to stroke were approximately 41 per 100,000 in 2000–2002 [4], and in 2005, stroke was responsible for over 90,000 deaths in Brazil [3]. Improvements in health care programs and a decrease in tobacco consumption have most likely influenced a decline in the rates of stroke in recent decades [1,5]. Diabetes has affected mortality as an associated cause, although deaths directly attributable to diabetes have remained relatively stable in recent decades [1,6]. Diabetic patients in Brazil have a more than three-fold excess mortality rate compared to the general population [7,8] and, as in other settings, most of this excess mortality is driven by increased cardiovascular mortality risk [7].

In addition to its effect on mortality, diabetes mellitus is strongly correlated with physical limitations and functional disability [9-13]. Individuals with diabetes are about two to three times more likely to have a disability than those without [14,15]. Individuals with diabetes mellitus also report a greater number of limitations with regard to basic and instrumental activities of daily living [13]. Longitudinal studies have found that individuals with diabetes are more likely to become functionally disabled [13,14,16-18] and less likely to transition from a disabled to disability-free status [13,19,20].

The combined effect of diabetes and stroke on disability has been previously studied [11,21,22], but remains largely unexplored in Brazil and Latin America. Studies have indicated that a combined history of diabetes and stroke is strongly associated with disability and quality of life [11,21,22], even though there have been contradictory findings in terms of the effect on mortality. Recently, studies in Brazil have highlighted the necessity of controlling for associated comorbidities such as cardiac disease and stroke to better evaluate the disabling effects of diabetes among the elderly [23]. Two cross-sectional studies in different regions of Brazil found that cardiovascular disease [23] and stroke [24] were strongly correlated with measures of functional status. Although diabetes was among the variables included in those studies, its effects on functional limitations were either not statistically significant [23], or only associated with specific activities of daily living, such as household tasks and mobility [24].

Given the limited availability of longitudinal data in developing countries, most studies that analyzed the impact of diabetes mellitus and/or stroke on functional status have used cross-sectional data. Few studies, mostly those conducted in developed countries, have evaluated health transitions associated with these chronic conditions [11,21,22]. The current study addresses the gap in knowledge about the combined effects of diabetes and stroke on the transitions in functional status and the health of elderly populations in São Paulo, the largest city in Brazil.

## Methods

### Data

This study analyzes data from two waves of the Survey on Health and Well-being of the Elderly (Salud, Bienestar y Envejecimiento en América Latina y el Caribe Proyecto, SABE) conducted in São Paulo, Brazil. SABE is a multi-center survey with respondents in seven capital/major cities throughout Latin America and the Caribbean, and it investigates the health and well-being of older adults (age 60 and over). The Institutional Review Board (IRB) at the School of Public Health in São Paulo University (Universidade de São Paulo) has approved the study protocol in Brazil. The study has also been approved by the IRB at the University of Illinois at Urbana-Champaign. Participants provided consent to have their data used for research purposes.

The baseline sample in São Paulo was obtained using a two-stage stratified sampling process based on the 1995 National Household Survey master sampling frame. Individuals age 75 and over were oversampled. Data in the first wave were collected in two stages. The first stage consisted of a household interview conducted by a single interviewer using a standardized questionnaire that included several questions about the living conditions and health status of the older adult. The second stage of data collection included a household visit by a pair of interviewers who completed anthropometric and physical-performance measurements. Data in the first wave were collected during 2000 and the first quarter of 2001. In the baseline wave, response rates reached 84.6% in São Paulo. The first stage contains information on 2,143 individuals. Additional characteristics of the baseline data collection process have been described elsewhere [25].

Data for the second wave were collected in 2006 by the São Paulo researchers as a follow-up to the 2000 baseline survey. They used mortality data from the Fundação Sistema Estadual de Análise de Dados (the SEADE foundation), which analyzes relevant social, demographic and economic data in the São Paulo state, and the Programa de Aprimoramento das Informações de Mortalidade (Program of Improvement of Information on Mortality, PRO-AIM), which collects and organizes mortality data for the São Paulo municipality, to identify subjects who had died between 2000 and 2006; the researchers did this via a search based on the names, sex, dates of birth and addresses listed in the 2000 database.

Trained interviewers visited the addresses and neighborhoods of surviving participants from the 2000 survey to re-establish contact. For those not found during these visits, interviewers used additional contact information collected in the baseline survey (e.g., telephone numbers of children or other relatives) to obtain information about their current location. The 2006 questionnaire was very similar to the 2000 questionnaire, but included additional questions that complemented the previous study. In both years, respondents provided information about possible limitations on ADL measures and assistance required to perform these activities. Questions related to ADL measures and assistance were the same in the baseline and the 2006 follow-up [26].

Of the 2,143 participants in the first wave of SABE São Paulo, 89 (4.15%) individuals had missing information on selected variables. There are no age or sex differences between those with missing and complete data. The final sample includes 2,054 individuals with baseline data on selected variables.

In the baseline survey, 373 participants (18.22%) reported being previously diagnosed with diabetes, and 162 (7.12%) reported being previously diagnosed with stroke. In this study, subjects were classified into four groups: (1) subjects with neither diabetes nor stroke (n = 1,562); (2) subjects with diabetes but no stroke (n = 330); (3) subjects with stroke but no diabetes (n = 119); and subjects with diabetes and stroke (n = 43).

### Statistical methods

Prevalence rates were obtained using STATA 12.1 SE for Windows (StataCorp, College Station, TX). Sample weights were used in the calculation of summary statistics, including means and percentages. Logistic regression models were used to assess the relationship between selected variables and the prevalence of activity of daily living (ADL) limitations, severe ADL limitations and requiring ADL assistance. Multivariate multinomial regression models were used to address health transitions. For those without disabilities at the baseline, the following transitions were measured: incidence (disability-free to disabled); mortality (disability-free to dead); and retention status for individuals who reported being healthy (disability-free) in both waves. For those with a disability at the baseline, the following transitions were measured: recovery (disabled to disability-free); mortality (disabled to dead); and retention status for participants who reported having a disability in both waves. Finally, an additional transition was included for those who were disability-free or disabled at baseline but who lacked information in the second wave, due to either loss in the follow-up or missing data on the disability measure. Similar models were used for severe ADL limitations and requiring ADL assistance. Odds-ratios and relative risk ratios for each outcome are provided along with the corresponding 95% confidence intervals. Control variables included age, sex, education, selected medical conditions, smoking and marital status. A significance level of 0.05 was chosen for all tests.

### Measures

This study considered sociodemographic characteristics of respondents, including age at baseline; gender (male or female); level of education (illiterate, defined as having no education, and literate, defined as one or more years of education); and marital status (married or unmarried). Participants also declared whether they had ever smoked and how they rated their health. We compared those who reported fair/poor health to those who reported good/very good/excellent health.

Diagnosed health conditions were obtained via self-report in SABE. In the baseline survey, individuals were asked if a physician had told them that they had diabetes and/or stroke. Other self-reported health conditions included heart disease, high blood pressure, cancer and arthritis; we created an index that included these four health conditions.

Self-reported limitations on the following six activities were considered for the ADL measure: dressing, bathing, eating, getting into and out of a bed (transferring), using the toilet and getting across a room. Individuals were given the following introduction: “Here are a few everyday activities. Please tell me if you have any difficulty with these because of a health problem. Exclude any difficulties you expect to last less than three months.” Following this introduction, they were asked, “Do you have difficulty …?” The possible answers for each one of the six ADL items were “yes,” “no,” “does not know” and “no response.” Those who answered “does not know” or who did not provide a response were classified as missing. Individuals with difficulties in at least three ADL items were categorized as having severe ADL limitations. In addition, one measure captured the extent of assistance required to perform basic life activities. Individuals were asked whether a spouse or another person assisted them in conducting basic daily activities. Those who reported receiving assistance with at least one ADL activity were categorized as receiving help with ADL activities.

## Results

Table 1 presents selected sociodemographic and health characteristics of the sample by diabetes/stroke status. Among respondents, 76.4% reported not having been previously diagnosed with either diabetes or stroke (group 1); 16.5% reported having only diabetes (group 2); 5.4% reported having only a stroke (group 3); and 1.8% reported having both diabetes and stroke (group 4). About half of the respondents (47.9%) had a history of smoking, and 53.8% rated their health “fair” or “poor.” The prevalence of ADL limitations reached 19.0% in the sample; 5.6% of respondents had severe ADL limitations and 7.4% received ADL assistance. Comparisons between the four groups showed no differences with regard to age, marital status or household arrangements. However, there were differences in gender; educational level; incidence of high blood pressure and heart attack; reporting fair/poor health; having ADL limitations; having severe ADL limitations; and receiving assistance with ADL. Men were more likely than women to report having experienced a stroke but no diabetes. The prevalence of high blood pressure and heart disease was lowest among those who had not experienced stroke and diabetes. In addition, having diabetes and/or stroke clearly impacted older adults’ perceptions of their health as poor or fair. Those who reported having a stroke and those having both diabetes and stroke were more likely to report having ADL limitations, having severe ADL limitations and receiving ADL assistance.

**Table 1:** Characteristics at baseline by diabetes and stroke status, SABE, São Paulo, 2000 (weighted estimates)

	**Total**		**No diabetes, no stroke**		**Diabetes, no stroke**		**Stroke, no diabetes**		**Diabetes and stroke**		
	**(n=2054)**	**%**	**(n=1562)**	**%**	**(n=330)**	**%**	**(n=119)**	**%**	**(n=43)**	**%**	**p-value**^**a**^
Age group (years)											0.398
60-74	1102	78.1	838	78.1	186	80.0	57	73.3	21	78.6	
75+	952	21.9	724	21.9	144	20.0	62	26.7	22	21.4	
Gender											0.033
Male	845	41.5	638	41.0	124	39.2	62	54.7	21	41.8	
Female	1209	58.5	924	59.0	206	60.8	57	45.3	22	58.2	
Marital status											0.392
Married	1082	57.4	810	57.1	182	58.5	63	55.5	27	67.0	
Unmarried	972	42.6	752	42.9	148	41.5	56	44.5	16	33.0	
Level of education											0.023
Illiterate	509	21.4	369	20.2	84	21.8	41	33.8	15	32.9	
Literate	1545	78.6	1193	79.8	246	78.2	78	66.2	28	67.1	
Never smoked	1106	52.1	846	52.3	184	52.8	53	47.0	23	51.4	0.195
High blood pressure	1107	53.5	736	47.3	251	74.2	84	68.7	36	82.3	<0.001
Had heart attack	444	19.7	299	17.0	86	25.7	46	39.3	13	19.1	<0.001
Had cancer	75	3.3	54	3.2	14	3.7	7	5.0	0	0.0	0.283
Had arthritis	691	32.3	526	32.0	118	35.3	31	25.3	16	37.9	0.264
Fair/poor health	1133	53.8	783	48.1	228	70.1	89	76.7	33	75.5	<0.001
ADL limitations	483	19.0	323	16.4	73	19.3	58	40.8	29	61.8	<0.001
Severe ADL limitations	169	5.6	89	3.5	22	5.5	36	24.7	22	41.9	<0.001
Receives assistance with ADL	205	7.4	113	5.2	25	6.2	43	28.3	24	48.5	<0.001

^a^ χ^2^ statistical test.

Table 2 presents results of unadjusted and adjusted logistic regressions predicting the prevalence of ADL limitations, severe ADL limitations and receiving ADL assistance in the baseline survey across the four groups. For each of the measures, the presence of diabetes did not increase the risk of limitations and assistance *per se*, but it did increase the risk when experienced in combination with stroke. After controlling for demographic, social and health conditions, individuals with stroke but not diabetes were 3.4 times more likely to have ADL limitations, and this already elevated risk more than doubled for those suffering from both diabetes and stroke (OR 7.34, 95% CI 3.73-14.46). Similar effects were observed for severe ADL limitations and receiving ADL assistance. Being older (75+ years) and having two or more health conditions increased the likelihood of having ADL limitations and severe ADL limitations as well as receiving ADL assistance. Older women were 37% more likely than men to have ADL limitations (95% CI, 1.04-1.81; gender differences for severe ADL limitations and receiving ADL assistance were not statistically significant). Individuals who were literate were less likely to have ADL limitations (OR 0.73, 95% CI 0.58-0.94) and to receive ADL assistance (OR 0.5, 95% CI 0.36-0.70) than those who were illiterate.

**Table 2:** Logistic regression coefficients, Brazil, 2000-2006

**Variables**	**Unadjusted**			**Adjusted**		
	**OR**	**95% CI**	**p-value**	**OR**	**95% CI**	**p-value**
**ADL limitations**						
Diabetes and stroke status						
Diabetes only	1.09	[0.82,1.45]	0.5584	0.96	[0.71,1.30]	0.8137
Stroke only	3.65	[2.49,5.33]	<0.0001	3.37	[2.26,5.04]	<0.0001
Diabetes and stroke	7.95	[4.15,15.21]	<0.0001	7.34	[3.73,14.46]	<0.0001
Age (75+)				2.46	[1.95,3.09]	<0.0001
Female				1.37	[1.04,1.81]	0.0276
Literate				0.73	[0.58,0.94]	0.0129
Married				0.96	[0.76,1.22]	0.7547
Never smoked				0.94	[0.73,1.21]	0.6323
Health conditions						
One				1.46	[1.08,1.96]	0.0132
Two or more				2.32	[1.73,3.13]	<0.0001
**Severe ADL limitations**						
Diabetes and stroke status						
Diabetes only	1.18	[0.73,1.92]	0.4966	1.15	[0.70,1.89]	0.5858
Stroke only	7.18	[4.60,11.21]	<0.0001	6.93	[4.27,11.22]	<0.0001
Diabetes and stroke	17.34	[9.19,32.72]	<0.0001	19.75	[9.81,39.76]	<0.0001
Age (75+)				4.00	[2.68,5.97]	<0.0001
Female				1.14	[0.73,1.76]	0.5647
Literate				0.55	[0.38,0.79]	0.0011
Married				0.72	[0.49,1.06]	0.0946
Never smoked				0.86	[0.58,1.28]	0.4567
Health conditions						
One				1.21	[0.74,1.97]	0.4434
Two or more				1.56	[0.96,2.52]	0.0715
**Assistance with ADL**						
Diabetes and stroke status						
Diabetes only	1.05	[0.67,1.65]	0.8284	0.99	[0.62,1.57]	0.9543
Stroke only	7.26	[4.77,11.04]	<0.0001	6.72	[4.28,10.56]	<0.0001
Diabetes and stroke	16.20	[8.61,30.46]	<0.0001	16.57	[8.39,32.73]	<0.0001
Age (75+)				3.11	[2.19,4.42]	<0.0001
Female				0.97	[0.65,1.44]	0.8637
Literate				0.50	[0.36,0.70]	<0.0001
Married				0.75	[0.53,1.07]	0.1111
Never smoked				0.93	[0.65,1.33]	0.6766
Health conditions						
One				1.45	[0.92,2.27]	0.1079
Two or more				1.85	[1.18,2.89]	0.0072

Baseline categories: no diabetes and no stroke, 65-74 years old, male, illiterate, unmarried, has smoked, no additional health conditions.

Between 2000 and 2006, 624 individuals remained free of ADL limitations, 242 developed ADL limitations, 114 remained ADL limited, 81 recovered from ADL limitations, 603 died and 392 were lost in the follow-up. Results of a multinomial logistic regression (shown in Table 3) further confirmed the combined effect of diabetes and stroke on health transitions (i.e., the incidence of, continuity of, and recovery from limitations, as well as the incidence of death) between 2000 and 2006. Having diabetes by itself was not associated with becoming or remaining ADL limited or recovering from ADL limitations between waves. However, diabetes was marginally associated with higher mortality (RRR 1.47, p-value = 0.063). Those who reported having had a stroke were 2.6 times more likely to develop ADL limitations, but this risk increased to almost 4 times higher among those with both stroke and diabetes. Stroke was also associated with a higher risk of remaining disabled (RRR 4.28, 95% CI 1.53-11.95) as well as higher mortality (RRR 3.42, 95% CI 1.65-7.09), and its association with diabetes further increased these risks. For example, those with diabetes who had suffered a stroke were 12.8 times more likely to remain disabled and 4.4 times more likely to die between waves. As expected, increases in age were associated with mortality as well as higher risks of becoming and remaining ADL limited. Women were more than twice as likely as men to become and remain ADL limited, but were less likely to die between waves (RRR 0.66, 95% CI 0.44-0.98). This result is consistent with older women’s greater life expectancy in São Paulo vis-à-vis their male counterparts [25]. Having additional health conditions increased the risk of developing ADL limitations, remaining limited or dying. Those who were literate were less likely to remain ADL limited and faced lower mortality rates, compared to those with no education. Those who reported having never smoked had lower mortality. Individuals who were older, female, or living alone and those who reported stroke or additional health conditions in the baseline survey were more likely to be lost in the follow-up period. Results for severe ADL limitations (available upon request) confirm that stroke is also associated with a higher incidence of these limitations (RRR 5.2, 95% CI 2.1-12.5). Both alone and in association with diabetes, stroke increased the risk of remaining severely ADL limited and of dying. Older age was associated with a higher incidence of severe ADL limitations, remaining severely ADL limited and dying. Having additional health conditions was also associated with a higher incidence of severe limitations. Finally, results for receiving ADL assistance (available upon request) confirmed that having suffered a stroke, both by itself and in combination with diabetes, was associated with a higher likelihood of receiving ADL assistance; however, there was no statistical association for diabetes only. Results by gender were not statistically significant, but increased age was associated with higher levels of requiring assistance. Those older than 75 years were over six times more likely than those in the younger group to start receiving personal assistance in the second wave (RRR 6.6, 95% CI 3.7-12.0), and were approximately six times more likely to continue using personal assistance if they already needed help in the baseline wave (RRR 5.9, 95% CI 2.2-15.8).

**Table 3:** Multinomial logistic regression coefficients, Brazil, 2000-2006

**Variables**	**RRR**	**95% CI**	**p-value**
**Incidence of ADL limitations**			
Diabetes and stroke status			
Diabetes only	1.03	[0.66,1.63]	0.8893
Stroke only	2.65	[1.11,6.33]	0.0287
Diabetes and stroke	3.97	[0.70,22.59]	0.1183
Age (75+)	2.80	[1.97,3.98]	<0.0001
Female	2.11	[1.33,3.34]	0.0020
Literate	0.73	[0.44,1.22]	0.2202
Married	1.00	[0.71,1.43]	0.9842
Never smoked	0.91	[0.59,1.40]	0.6722
Health conditions			
One	1.71	[1.13,2.58]	0.0123
Two or more	2.90	[1.93,4.34]	<0.0001
**Remained ADL limited**			
Diabetes and stroke status			
Diabetes only	0.97	[0.54,1.76]	0.9307
Stroke only	4.28	[1.53,11.95]	0.0063
Diabetes and stroke	12.84	[2.95,55.99]	0.0010
Age (75+)	3.82	[2.34,6.24]	<0.0001
Female	2.44	[1.31,4.54]	0.0057
Literate	0.49	[0.28,0.85]	0.0118
Married	1.45	[0.78,2.71]	0.2366
Never smoked	0.80	[0.44,1.43]	0.4380
Health conditions			
One	3.42	[1.61,7.27]	0.0019
Two or more	7.78	[3.79,15.97]	<0.0001
**Recovered from ADL limitations**			
Diabetes and stroke status			
Diabetes only	0.94	[0.45,1.98]	0.8760
Stroke only	0.40	[0.07,2.27]	0.2936
Diabetes and stroke	6.77	[1.07,42.87]	0.0425
Age (75+)	0.92	[0.47,1.78]	0.7940
Female	1.47	[0.77,2.82]	0.2423
Literate	0.71	[0.39,1.32]	0.2733
Married	0.79	[0.41,1.55]	0.4932
Never smoked	1.03	[0.60,1.77]	0.9227
Health conditions			
One	1.79	[0.86,3.72]	0.1180
Two or more	2.92	[1.45,5.86]	0.0033
**Mortality**			
Diabetes and stroke status			
Diabetes only	1.47	[0.98,2.21]	0.0616
Stroke only	3.42	[1.65,7.09]	0.0014
Diabetes and stroke	4.37	[1.15,16.56]	0.0305
Age (75+)	6.15	[4.59,8.23]	<0.0001
Female	0.59	[0.40,0.88]	0.0097
Literate	0.58	[0.41,0.83]	0.0031
Married	0.76	[0.54,1.06]	0.1001
Never smoked	0.60	[0.43,0.82]	0.0022
Health conditions			
One	2.03	[1.44,2.86]	0.0001
Two or more	2.89	[1.99,4.18]	<0.0001
**Lost in follow-up**			
Diabetes and stroke status			
Diabetes only	0.82	[0.53,1.27]	0.3621
Stroke only	1.98	[1.04,3.77]	0.0388
Diabetes and stroke	1.00	[0.19,5.36]	0.9965
Age (75+)	1.63	[1.16,2.29]	0.0056
Female	1.50	[1.09,2.05]	0.0130
Literate	1.06	[0.66,1.69]	0.8061
Married	0.83	[0.57,1.21]	0.3295
Never smoked	0.75	[0.54,1.05]	0.0887
Health conditions			
One	1.52	[1.08,2.14]	0.0183
Two or more	1.59	[1.12,2.24]	0.0096

Baseline categories: no diabetes and no stroke, 65-74 years old, male, illiterate, unmarried, has smoked, no additional health conditions.

## Discussion

Our main results show that the impact of diabetes on disability manifests only in combination with stroke. Older adults with diabetes who had experienced a stroke were more likely to have ADL limitations, severe ADL limitations and higher mortality risks than those with neither condition. These respondents were also more likely to develop a disability or remain disabled over a six-year period. While having a stroke alone was a risk factor, the combination with diabetes intensified the risks of having and developing a disability as well as mortality risks. These findings expose the major burden that co-existing health conditions place on ability and quality of life among older adults. As discussed by Otiniano and colleagues, patients recovering from a stroke, especially those with diabetes, should receive additional attention upon discharge to the community, given their greater disability and mortality risks [11].

Second, our study confirmed that older women in São Paulo face greater risks than older men of being, becoming and remaining ADL limited. These results contribute to a growing discussion about the greater disability burden and lower quality of life in later years among women in Brazil [25,27-31]. Women constitute the majority of the elderly population in São Paulo, and their higher disability risks reduce their chances of maintaining social and economic autonomy. Our results showed no significant association between receiving ADL assistance and gender. These results contrast with previous findings that women’s needs for personal care assistance went unmet for a greater proportion of time that they lived with a disability compared to men [25]. This discrepancy is related to the effects of additional control variables included in the current study. Socioeconomic and additional health variables were included in addition to age—the only control variable in the previous study.

These new findings highlight the ways in which women’s lower socioeconomic and health status influence the level of care and assistance they receive in later years. First, higher rates of illiteracy among older women in São Paulo (29% of women are illiterate compared to 19% of men) likely indicate lower socioeconomic status, which limits older women’s access to health care services in general and personal assistance in particular. Older women’s marital status and longer life expectancies can also explain their lower chances of receiving assistance in their later years [25,32]. In São Paulo, older women are more likely than older men to be unmarried or widowed (63% and 25% for women and men, respectively), and they are also more likely to have additional chronic conditions (1.23 additional conditions among women compared to 0.96 among men, p < 0.001). Thus, a common scenario for an older woman in São Paulo is to find herself living alone with scarce economic and social resources to attend to her needs for care in later life. This is a rather inequitable outcome for Brazilian women who are, as in most countries, the majority of informal and long-term caregivers within their families and communities [33-35].

Our results confirm that the oldest among the old (75+ years), who are more likely to suffer from multiple health conditions, are at a higher risk of having ADL limitations and requiring ADL assistance. Within a six-year period, the oldest Brazilians were also more likely to develop ADL limitations, to remain ADL limited and to die. The implications of these results are particularly serious, given the low probabilities of recovery from disability observed in this study and the rapid increase of the elderly population in Brazil, particularly the oldest of the old.

The persistence of CNC diseases such as stroke and diabetes represents a major economic burden for the Brazilian state and society. Recent reports estimated that the labor force reduction tied to diabetes, heart disease and stroke would represent a loss of over USD $4 billion in economic outputs for the country between 2006 and 2015 [1,36]. The annual costs associated with hospitalization of Brazilians with diabetes are estimated at USD $264 million [37], and an additional USD $327 million are needed to provide acute treatment for incident stroke [38]. Unpaid assistance and care services provided by families, along with high-cost health services, is yet another dimension of the economic and social burden of CNC diseases in Brazil.

Our results on the impact of education on disability are rather encouraging. Being literate reduced the risk of having and retaining ADL and severe ADL limitations and dying. These findings improve the scholarly understanding of the influence socioeconomic indicators such as education have on the health conditions of populations in less-developed countries. The association between lower socioeconomic conditions, higher mortality and lower healthy life expectancies is well established in developing nations [39]. In Brazil, existing evidence based on cross-sectional data indicates similar patterns [40,41]. Camargos and colleagues found that older adults with five years of schooling or more spent a higher proportion of their lives without disability than those with less education [28].

Smoking is a major risk factor associated with CNC diseases such as stroke, and tobacco consumption in Brazil is widespread despite recent declines. Our results showed the positive health outcomes of not smoking, particularly reduced mortality risk. Recent health policy discussions have highlighted improvements in cardiovascular and chronic respiratory disease mortality rates in Brazil, which are partially attributable to declines in tobacco use and greater access to primary care [1]. Nonetheless, the direction of smoking trends in Brazil remains ambiguous, with some evidence showing steady rates of smoking between 2006–2009 [1].

The Brazilian state’s efforts to control tobacco use are part of a broader comprehensive strategy that has prioritized CNC diseases in Brazil’s public health policy, especially since 2006 [1]. Some notable measures include the provision of health care; programs to increase awareness of healthy diets and promote physical activity; the control of alcohol consumption; and the widespread delivery of drugs to those at high risk of cardiovascular diseases [1]. The universalization of health care in Brazil is at the core of these important policy developments. Recent studies evaluating the Brazilian public health strategy have emphasized the need to shift the current acute-care-oriented health care model to a chronic-care model to meet the rising burden of chronic conditions resulting from demographic and epidemiological transformations in Brazil [1,42]. One premise of this model is the need to build an integrated and inter-sectorial health care system with the goal of providing cost-effective interventions to maximize health outcomes. In general, policy recommendations by Brazilian organizations, as well as international organizations such as the World Health Organization, suggest health education and awareness programs as effective strategies for linking health care and education in the prevention of CNC diseases. As Brazil anticipates a growing number of individuals with chronic conditions, expanding efforts to improve health care access and education levels can multiply the impact on CNC disease prevention and lead to a reduction in economic and social costs.

### Limitations

Self-reported data could potentially be a source of bias. However, methodological studies have shown that self-reported data on functional disability correspond to medical diagnosis [43]. The use of self-reported health conditions is also problematic. This study is not able to take into account the increased disability and mortality of individuals with undiagnosed conditions; therefore, the estimates presented here are conservative estimates of the real burden faced by this population. However, undiagnosed rates tend to decrease with age, which reduces the bias in this study of older adults [27]. In addition, the results provided in this study might not be representative of the older population residing in institutions, given the methodological design of the SABE survey that focused in non-institutionalized population in the baseline. Nonetheless, studies have estimated that the institutionalized population in Brazil is relatively small [44], thereby minimizing the potential for bias in our results. Our analyses did not control for income, which can influence the probability of receiving personal assistance. However, many older women in São Paulo do not have personal income, which is why many studies on Brazil use education as a measure of socioeconomic status [28]. Finally, our results reflect the experiences of older adults in the largest city in Brazil, which may differ from the general Brazilian population.

## Conclusions

This is the first study to use longitudinal data to generate estimates of the joint impact of diabetes and stroke on mortality and disability, as well as transitions over time among older adults in São Paulo, Brazil. The study also provides analysis and discussion of gender and socioeconomic status differences in receiving ADL assistance as well as the impacts of smoking habits on mortality as a major risk factor for CNC diseases such as diabetes and stroke. The results of our study are useful for the development of public policy strategies that target a growing segment of the Brazilian elderly population affected by chronic diseases and disabilities and with pressing needs for specialized care services. Our discussion highlights the positive effects of education in reducing the burden of CNC diseases and their related social and economic costs for Brazilian society. This discussion is highly relevant in the current context of the rising obesity and diabetic epidemics affecting Brazil today.

## Competing interests

The authors declare that they have no competing interests.

## Authors’ contributions

FCDA conceived, designed and coordinated the study; performed the statistical analysis; and participated in manuscript writing. PEG assisted with manuscript writing and participated in the interpretation of results and discussion. MLL and YAOD participated in the collection, analysis and interpretation of data. All authors read and approved the final manuscript.

## Pre-publication history

The pre-publication history for this paper can be accessed here:

http://www.biomedcentral.com/1471-2458/12/361/prepub
